# *Actinobacillus pleuropneumoniae* Serotypes by Multiplex PCR Identification and Evaluation of Lung Lesions in Pigs from Piedmont (Italy) Farms

**DOI:** 10.3390/ani14152255

**Published:** 2024-08-03

**Authors:** Matteo Cuccato, Sara Divari, Silvia Ciaramita, Alessandra Sereno, Domenico Campelli, Pier Giuseppe Biolatti, Bartolomeo Biolatti, Francesco Meliota, Enrico Bollo, Francesca Tiziana Cannizzo

**Affiliations:** 1 Department of Veterinary Sciences, University of Turin, Grugliasco, 10095 Turin, Italy; matteo.cuccato@unito.it (M.C.); sara.divari@unito.it (S.D.); silvia.ciaramita@edu.unito.it (S.C.); alessandra.sereno@unito.it (A.S.);; 2 Fatro S.p.A., Ozzano nell’Emilia, 40064 Bologna, Italy; 3 Independent Researcher, 10100 Turin, Italy; 4 Pollenzo Campus, University of Gastronomic Sciences, 12042 Pollenzo, Italy

**Keywords:** *Actinobacillus pleuropneumoniae*, porcine pleuropneumonia, swine, respiratory diseases, serotyping, multiplex PCR

## Abstract

**Simple Summary:**

Porcine pleuropneumonia, caused by *Actinobacillus pleuropneumoniae*, is one of the main causes of massive losses in the pig industry, with a high economic impact. This study aimed to identify *A. pleuropneumoniae* serotypes isolated from pneumonic lesions in dead pigs, naturally infected, in some Piedmontese farms and to describe the macroscopic and microscopic lesions. The identification of circulating serotypes in swine farming is crucial to adopting optimal strategies to control the diseases, such as vaccination plans and biosecurity measures.

**Abstract:**

Porcine pleuropneumonia (PPP) is one of the main causes leading to massive losses in the pig industry, with high economic impacts. Among different etiological agents, *Actinobacillus pleuropneumoniae* (APP) is responsible for severe fibrinous-necrotizing pleuropneumonia. A total of 19 different APP serotypes are currently recognized. This study aimed to identify APP serotypes isolated from pneumonic lesions in naturally infected and dead pigs in the Piedmont Region and to describe lesions. A total of 107 dead pigs with a suspected PPP diagnosis were included in this study. Lungs were evaluated using gross-pathology scoring systems, histopathology, and APP isolation and serotypes identification by multiplex PCR were conducted. Gross lung lesions were mainly represented by fibrinous pneumonia and pleuropneumonia. APP was isolated in 20/107 (18.7%) samples. PCR indicated APP DNA presence in 53/107 (49.5%) of lung samples. The most observed serotypes were serotype 2 in 24/53 (45.3%) and serotype 6 in 13/53 (24.5%) samples. Moreover, multiplex PCR results suggested a coinfection of different serotypes in five samples. This study emphasizes the importance of an integrated approach, utilizing various techniques, such as gross- and histopathology, and bacteriological culture and PCR, to enhance the diagnosis of APP infections.

## 1. Introduction

Porcine pleuropneumonia (PPP) is a respiratory infectious disease affecting pigs, caused by *Actinobacillus pleuropneumoniae* (APP) [1,2]. The economic impact is not only limited to the mortality rate (which can be as high as 10%) but also to treatment costs (elevated costs associated with antimicrobial treatments and vaccination plans). Average daily profit can be reduced by up to 34% while the deterioration of the feed conversion rate can reach 26% [3,4].

APP, a Gram-negative facultative anaerobic bacterium [5], is categorized into two biovars based on NAD metabolism: NAD-dependent biovar 1 and NAD-independent biovar 2 [6]. Serotyping, conversely, is predicated on the differential expression of capsular polysaccharide and lipopolysaccharide antigens [7,8], with 19 distinct serotypes identified thus far within biovar 1 or 2 [9].

PPP manifests in various clinical presentations: peracute, acute, and chronic [10]. In peracute cases, pigs succumb suddenly to systemic shock symptoms [8], while the acute presentation is characterized by respiratory distress, fever, and anorexia [8,10]. Chronic infections often lack clinical signs, with APP typically confined to tonsil crypts [7,10]. Necropsies commonly reveal hemorrhagic and/or fibrinous pleuropneumonia with areas of pulmonary consolidation in APP-infected pigs [11]. The presentation and severity of the disease depends on host factors, pathogen strain-related pathogenicity, and environmental and management factors [8]. However, limited information exists regarding gross lesion presentation among different serotypes and potential variations therein. Etiological diagnosis typically involves isolation using a blood agar medium in microaerophilic conditions, co-cultured with *Staphylococcus aureus* [1,5]. Yet, APP isolation can be challenging due to its unique culture conditions, and confirmation is sometimes elusive [12]. Confirmation methods include end-point PCR with specific primers for the *apxIV* gene, distinguishing APP from other *Actinobacillus* species [13]. Alternatively, matrix-assisted laser desorption/ionization-time of flight mass spectrometry (MALDI-TOF) can also be employed for confirmation purposes [2,14]. Despite the advantages of MALDI-TOF technology in expediting pathogen identification, challenges remain in bacteriological growth, prompting the search for a faster and more cost-effective alternative for detecting APP. Stringer and colleagues have proposed a culture-free APP detection method through PCR without bacteriological isolation, representing a valuable attempt to streamline etiological and serotype identification [12].

At the farm level, APP is routinely monitored through vaccination plans [8,15]. Commercial vaccines, comprising inactivated bacteria, purified toxoids, or a combination thereof [15], exhibit appropriate safety and efficacy. However, cross-protection against various serotypes is not consistently guaranteed and depends on the specific commercial product [15]. In Italy, historical control of APP outbreaks involves vaccination plans. Nevertheless, recent observations by veterinary practitioners indicate an increased mortality in pleuropneumonia syndromes attributed to APP. Notably, the circulation of APP serotypes in Italy has not been investigated, representing a gap in evaluating the efficacy of vaccination plans.

Therefore, the main objective of this study was to determine the circulating serotypes of APP in swine farms in Piedmont. To achieve this, samples were collected from deceased pigs with suspected APP infections.

## 2. Materials and Methods

### 2.1. Gross Evaluation and Sample Collection

**Figure 1:**
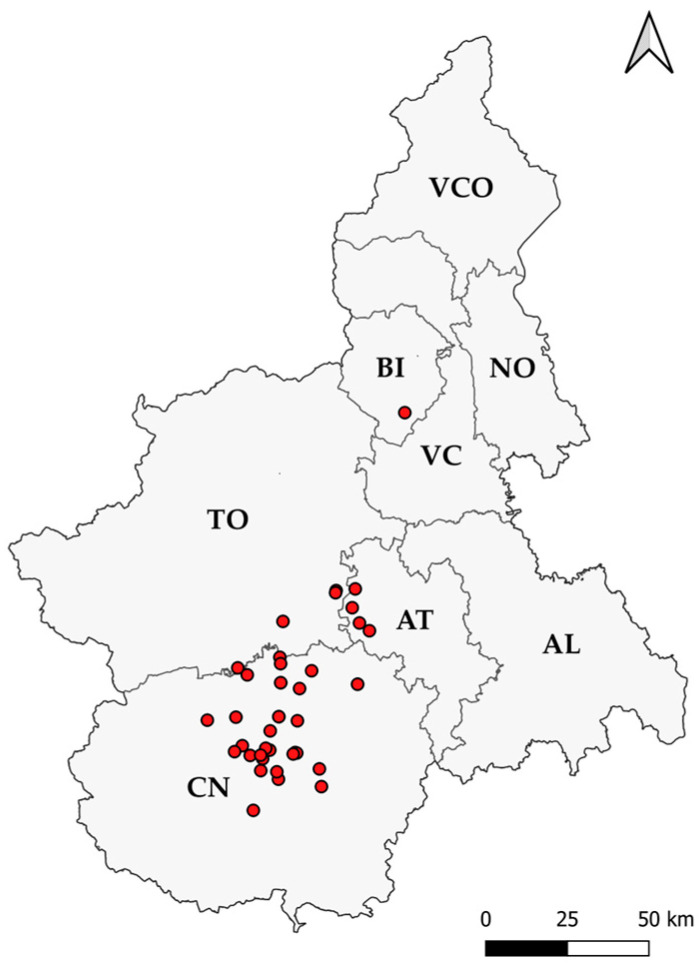
Pig farms distribution in Piedmont. Dead pigs were collected from a total of 40 pig farms, distributed in the provinces of Cuneo (CN), Asti (AT), Turin (TO), and Biella (BI). The other provinces are Alessandria (AL), Vercelli (VC), Novara (NO), and Verbania-Cusio-Ossola (VCO).

A total of 107 lungs belonging to dead pigs from 40 different farms in Piedmont (Italy) were collected from October 2020 to July 2023 (2 samples from each farm as minimum and 9 as maximum). The farms were located in different areas of the Piedmont provinces of Cuneo (CN), Asti (AT), Turin (TO), and Biella (BI). This area is of great importance for the pig farming system, representing one of the main and biggest in the Italian pork meat industry scenario. Their locations have been reported on a map (Figure 1). All farms were final stage production sites for pig fattening and, therefore, without any connections between them. Moreover, biosecurity measures were regularly adopted in all these farms.

Dead pigs with a differential diagnosis of PPP were selected by veterinary practitioners, and their lungs were sent to the Department of Veterinary Science in Turin for gross pathological evaluation and further investigations. Lungs were examined and sampled within a maximum of 6 h from the death (*n* = 87). When this time could not be respected, the lungs (*n* = 20) were collected from the pigs by the farm veterinarians and frozen at −20 °C.

Lung lesions were classified according to the list presented in Table 1, describing the main pulmonary lesions observed. The anatomo-pathological lesion categories used in the diagnostic protocol are catarrhal bronchopneumonia (CBP), purulent bronchopneumonia (PBP), interstitial pneumonia (IP), bronchopneumonia associated with interstitial pneumonia (BP + IP), fibrinous pneumonia or pleuropneumonia (FP or PP), interstitial pneumonia with oedema or polilobular pneumonia (IPP), pleuritis (PL), and pericarditis (PE). The lesion types were defined using the scheme proposed by Sørensen [16].

In addition, each pulmonary lobe (left cranial/medium/caudal lobes and right cranial/medium/accessory/caudal lobes) was assigned a score based on the extent of lobe involvement by lesions. The scoring system ranges from zero to four, providing a semiquantitative measure of lesion extent and severity (0: no lesion; 1: <25%; 2. 26–50%; 3: 51–75%; 4: >76%). The lung score was applied according to the modified methods of Madec and Derrien, and Madec and Kobish [17,18].

Finally, pleurisy lesions were also evaluated according to the SPES system (slaughterhouse pleuritis evaluation system), considering a 0–4 scale depending on the extent and location of the pleuritis, according to the method devised by Dottori and colleagues (2007) [19]. Briefly, the different scores were assigned according to the following observed lesions: 0—absence of pleural lesions; 1—cranioventral pleuritis and/or pleural adherence between lobes or at the ventral border of lobes; 2—dorsocaudal unilateral focal pleuritis; 3—bilateral pleuritis of type 2 or extended unilateral pleuritis (at least 1/3 of one diaphragmatic lobe); or 4—severely extended bilateral pleuritis (at least 1/3 of both diaphragmatic lobes).

After gross evaluation, sample collection for the microbiological and molecular investigations was performed. Briefly, the surface of the most representative lesion was disinfected with 70% ethanol before aseptically incising the area to collect a sample using a sterile swab. In particular, for microbiological investigation, swab samples were collected in the sub-pleural region after the dissection of the pleura from the parenchyma with a sterile blade. Moreover, on 59 examined lung samples, a piece of pulmonary parenchyma was collected and sent to an external laboratory for microbiological investigations. Finally, lung samples collected near the lesion were fixed in 4% neutral buffered formalin for histological investigations.

### 2.2. Histopathological Evaluation

Formalin-fixed lung samples were paraffin-embedded according to routine histological procedures. Representative sections of each sample were stained with hematoxylin and eosin (HE) stain for histological examination. All slides were observed with a Nikon Eclipse E600 light microscope (Nikon Corporation, Tokyo, Japan). The histological evaluation was conducted by examining a total of 5 HPF at 200× magnification for each sample.

### 2.3. APP Bacterial Isolation

Samples collected with sterile swabs were co-cultured with *Staphylococcus aureus* strain SS697 (ATCC no. 33862) on BD Columbia blood agar with 5% sheep blood (Becton Dickinson GmbH, Heidelberg, DE, Germany). The agar plates were incubated at 37 °C for 24 h in microaerophilic conditions with the addition of carbon dioxide using a Gaspak ez CO_2_ pouch system (Becton Dickinson GmbH, Heidelberg, DE, Germany). The colonies of APP clustered around *S. aureus* and produced a well-defined hemolysis zone. Isolated colonies were cultured in Brain Heart Infusion broth (BHI, Merk, KGaA, Darmstadt, DE, Germany), added with 0.01% of NAD (Merk), at 37 °C for 24 h in microaerophilic conditions as described above. The isolated APP strains were stored at −80 °C with a 30% glycerol solution. In addition, a total of 2 mL of inoculated BHI broth after 24 h was collected and centrifuged 14,000× *g* for 5 min. After removing the supernatant solution, the obtained pellet was further processed for APP species and serotype confirmation by multiplex PCR.

### 2.4. Microbiological Investigations

To further characterize the presence of porcine respiratory pathogens, microbiological investigations were conducted on 107 lung samples by an external laboratory. In particular, the presence of Porcine Respiratory and Reproductive Syndrome Virus (PRRSV), Circovirus, Swine Influenza Virus (SIV), and *Mesomycoplasma hyopneumoniae* were investigated by PCR according to the external laboratory’s routine methods. Moreover, bacteriological cultures were performed through non-selective conditions, and isolated bacterial colonies were identified by MALDI-TOF mass spectrometry according to the external laboratory’s routine methods.

### 2.5. DNA Extraction and Multiplex PCR

DNA was extracted from samples collected with sterile swabs and from APP strains cultured in BHI, using a QIAamp^®^ UCP Pathogen Mini kit (Qiagen, Hilden, DE, Germany), following the manufacturer’s instructions. DNA was quantified using a Nanodrop spectrophotometer (Thermo Fisher Scientific, Waltham, MA, USA). Two multiplex PCRs (APP-mPCR1 and APP-mPCR2) were performed as described in Stringer et al. [9]. In brief, a Qiagen multiplex PCR kit (Qiagen, Hilde, DE, Germany) was used for the APP-mPCRs, consisting of 12.5 μL of 2× Multiplex PCR Master Mix, 0.2 μM of each primer, 2.5 μL 10× CoralLoad gel tracking dye, and 2 μL DNA template in a final volume of 25 µL. The cycling conditions were 10 min at 95 °C, followed by 30 cycles of 95 °C for 15 s, 60 °C for 90 s, and 72 °C for 150 s. PCR products were visualized with 1.5% agarose gel, stained with MIDORI Green Advance DNA stain (Nippon Genetics Europe, Düren, DE, Germany), and visualized under UV. Genomic DNA of APP serotypes, provided by Dr. G. Alborali from the Istituto Zooprofilattico Sperimentale della Lombardia e dell’Emilia Romagna “Bruno Ubertini” in Italy, was included as a positive control. Nuclease-free water as a negative control was added in each PCR testing batch. The strains used as positive controls for the multiplex PCR have also been tested to validate the obtained results (Figure S1). APP DNA presence was confirmed by *apxIV* (423 bp) gene amplification, and specific products amplified by APP-mPCR1 and APP-mPCR2 were detected to identify serotypes of APP isolated or found in lung lesions [9]. APP biovar 2 was identified through a full-length functional *nadV* gene presence confirmed by a specific product at 1339 bp that is absent in biovar 1.

## 3. Results

### 3.1. Gross Evaluation

**Figure 2:**
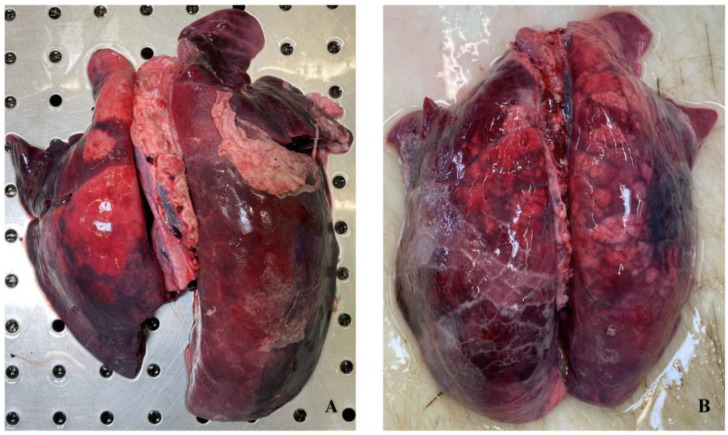
Pig lungs. Fibrinous pleuropneumonia (**A**). Bronchopneumonia is associated with interstitial pneumonia (**B**).

**Figure 3:**
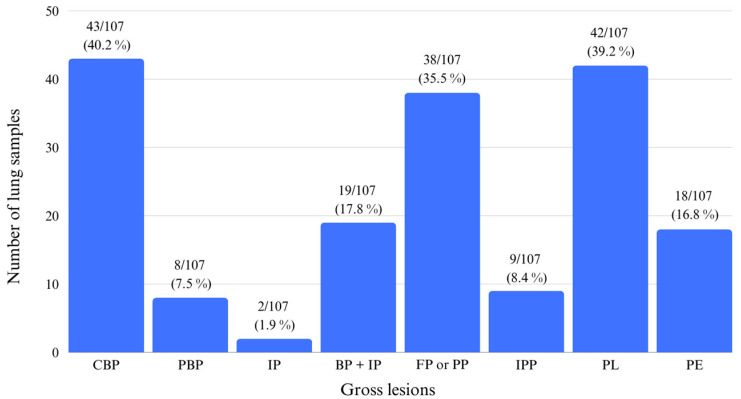
Pulmonary gross lesion distribution in lungs. Gross lesions legend: catarrhal pneumonia (CBP), purulent bronchopneumonia (PBP), interstitial pneumonia (IP), bronchopneumonia associated with interstitial pneumonia (BP + IP), fibrinous pneumonia or pleuropneumonia (FP or PP), interstitial pneumonia with oedema or polilobular pneumonia (IPP), pleuritis (PL), and pericarditis (PE).

Gross pulmonary lesions were mainly characterized by CBP in 43/107 (40.2%) samples, by PL in 42/107 (39.3%) samples, and by FP or PP in 38/107 (35.5%) samples (Figure 2). The remaining lesions were also observed, and their distributions can be visualized in Figure 3.

Average scores (mean ± sd) for each lobe are presented in Table 1. The highest average score was recorded for the right medium lobe (2.64 ± 1.32), followed by the right accessory lobe (2.42 ± 1.52). An explanation for the scoring classification is shown in Figure 2, reporting illustrative examples for each assigned score.

**Figure 4:**
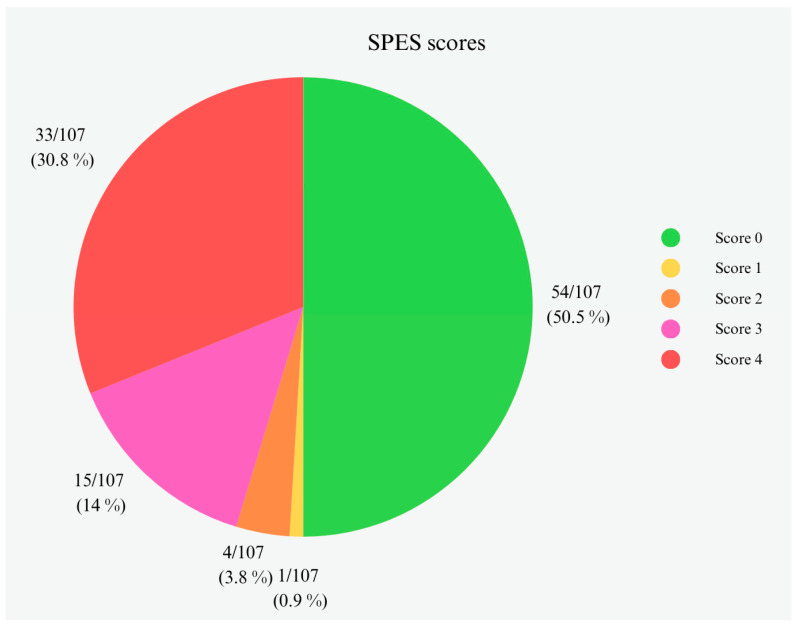
Pleurisy lesion distribution in the lungs according to the SPES system. Scores legend: 0—absence of pleural lesions; 1—cranioventral pleuritis and/or pleural adherence between lobes or at ventral border of lobes; 2—dorsocaudal unilateral focal pleuritis; 3—bilateral pleuritis of type 2 or extended unilateral pleuritis (at least 1/3 of one diaphragmatic lobe); and 4—severely extended bilateral pleuritis (at least 1/3 of both diaphragmatic lobes).

Finally, according to the SPES system, lung samples were classified with score 0 in 54/107 (50.5%) samples, score 1 in 1/107 (0.9%) samples, score 2 in 4/107 (3.8%) samples, score 3 in 15/107 (14%) samples, and score 4 in 33/107 (30.8%). The score distribution in the pig lungs can be observed in Figure 4. The average SPES score of this study was 1.74 ± 1.83.

### 3.2. Histopathological Evaluation

**Figure 5:**
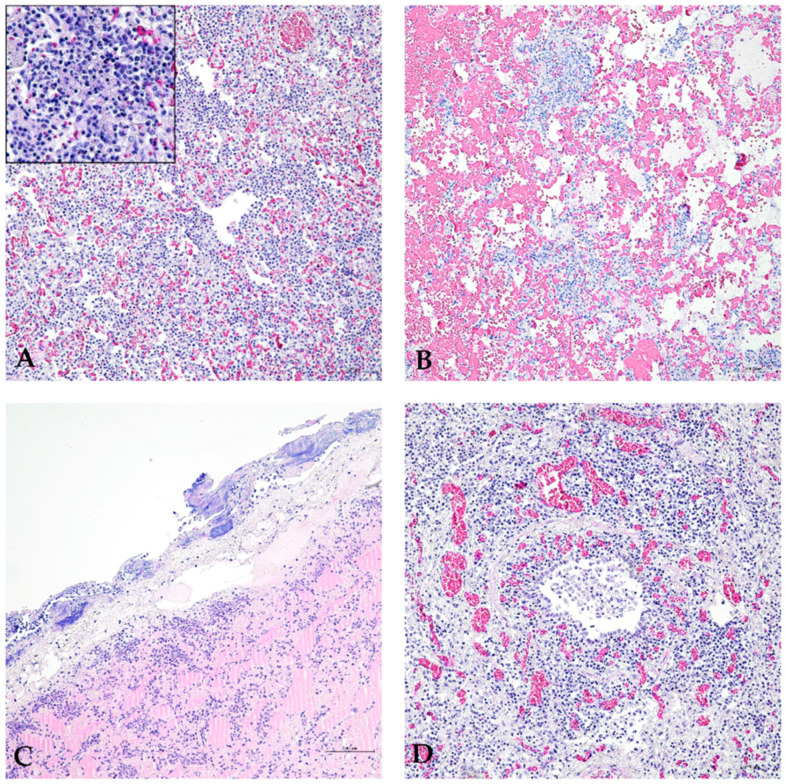
Pig lung histopathology. Inflammatory infiltrate ((**A**), HE, 200×): lymphocytes and neutrophils [insert of the inflammatory infiltrate (HE, 400×)]; congestion and hemorrhage with lymphocytic inflammatory infiltrate ((**B**), HE, 200×); pleural thickening with subpleural lymphocytic infiltrate and alveolar oedema ((**C**), HE, 200×); congestion with lymphocytic inflammatory infiltrate and exudate in the bronchiolar lumen ((**D**), HE, 200×).

HE-stained lung sections collected from pigs that died with a suspected diagnosis of PPP were evaluated, and each observed microscopic lesion was recorded. In particular, histopathological lesions of collected lungs were mainly described as inflammatory infiltrate of mixed leucocyte populations in 64/107 (59.8%) samples, congestion in 54/107 (50.5%) samples, pleural thickening in 53/107 (49.5%), hemorrhages in 53/107 (49.5%), exudate in the bronchiolar lumen in 43/107 (40.2%), and subpleural lymphocytic infiltrate in 43/107 (40.2%). Figure 5 shows an inflammatory infiltrate consisting of a leucocyte mixture (Figure 5A), congestion and hemorrhage (Figure 5B), severe pleural thickening with subpleural lymphocytic infiltrate (Figure 5C), and exudate in the bronchiolar lumen (Figure 5D). However, in 30/106 (28.3%) samples, other lesions were also observed, and their distributions are reported in Table S1.

### 3.3. Microbiological Investigations

APP colonies were morphologically identified as those clustered around *S. aureus* with a well-defined hemolysis zone. Therefore, APP bacteria was isolated in 20/107 (18.7%) samples.

Results of the microbiological investigations revealed the presence of at least one porcine respiratory pathogen in 68/107 (63.6%) lungs analyzed. Moreover, in 3/107 (2.8%) samples, only APP was identified, and in 16/107 (15%), no pathogens were identified (Table 2). In particular, APP serotypes were mainly found associated with PRRSV (31/107, 29%) and *M. hyopneumoniae* (11/107, 10.3%).

### 3.4. Serotypes Assignment of APP from Isolated Colonies and Lung Swabs by Multiplex PCR

**Figure 6:**
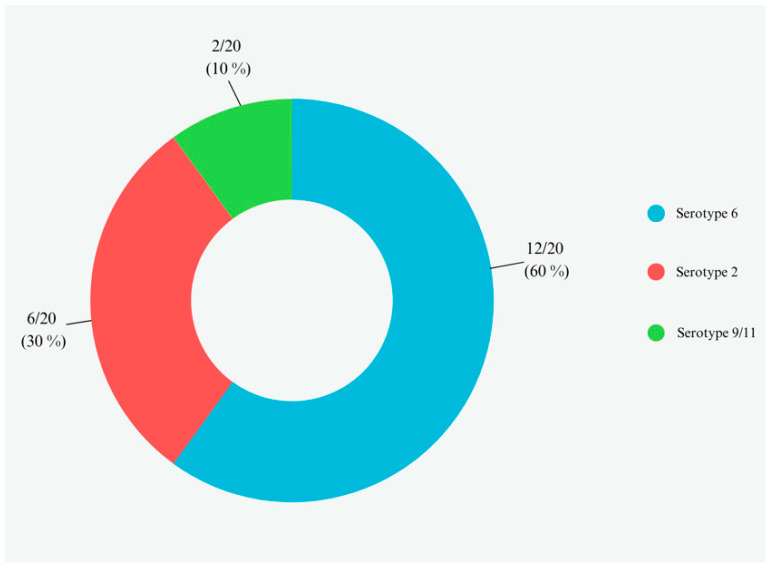
APP-cultured strains and serotype distribution. Serotypes of APP cultured from 20/107 (18.7%) lung swab samples.

APP-isolated strains were confirmed by PCR with the amplification of specific fragments consistent with the *apxIVA* gene (423 bp). Concerning serotyping results, the observed serotypes in cultured APP strains were serotype 2 with a specific fragment consistent with the *cps2E* gene (247 bp) in 12/20 (60%), serotype 6 with a specific fragment consistent with the *cps6F* gene (718 bp) in 6/20 (30%), and serotype 9/11 with a specific fragment consistent with the *cps9/11E* and *cps9/11F* genes (2105 bp) in 2/20 (10%) samples. Serotype distribution in cultured APP strains can be observed in Figure 6.

**Figure 7:**
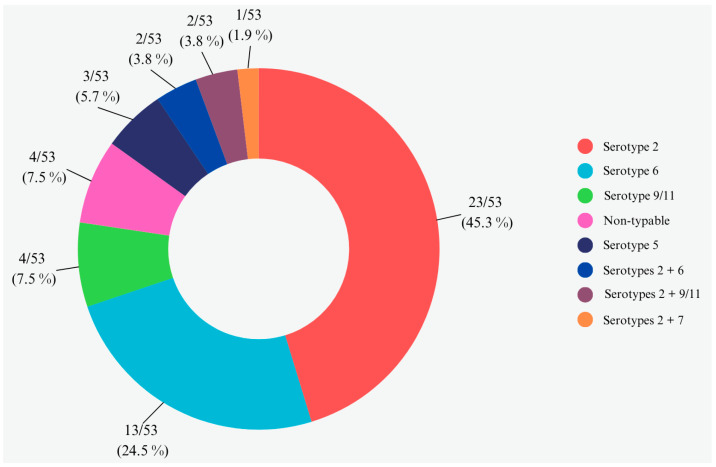
APP-positive samples and serotype distribution. Serotypes of APP were detected in 53/107 (49.5%) lung swab samples.

Furthermore, APP-mPCR1 and APP-mPCR2 were applied on DNA extracted from lung swabs. A specific fragment consistent with the *apxIVA* gene (423 bp) was observed in 53/107 (49.5%) lung swab samples, confirming the presence of APP in collected lungs. Regarding serotype assignment by the two multiplex PCR assays, the most observed serotypes were serotype 2 in 24/53 (45.3%) APP-positive samples and serotype 6 in 13/53 (24.5%) APP-positive samples. Furthermore, serotype 9/11 was observed in 4/53 (7.6%) APP-positive samples and serotype 5 with a specific fragment consistent with the *cps5B* gene (825 bp) in 3/53 (5.7%) APP-positive samples. Moreover, in 4/53 (7.6%) samples, the fragment relative to the *apxIVA* gene was only amplified without observing any other serotype-specific fragment. Specific fragments belonging to more than one serotype were also observed in addition to the specific fragment consistent with the *apxIVA* gene in the remaining five positive samples. In particular, in 2/53 (3.8%) samples, specific fragments consistent with the *cps9/11E* and *cps9/11F* genes were observed, as well as a specific fragment consistent with the *cps2E* gene; in 2/53 (3.8%) samples, a specific fragment consistent with the *cps6F* gene was observed, as well as aspecific fragment consistent with the *cps2E* gene; and in 1/53 (1.9%) samples, a specific fragment consistent with *cps7E* gene (601 bp) was observed, as well as a specific fragment consistent with the *cps2E* gene. Finally, the serotype distribution in our collected samples can be observed in Figure 7.

In addition, results on *nadV* gene amplification allowed us to distinguish between biovar 1 and 2. More specifically, 4/53 (7.6%) samples resulted in a specific fragment consistent with the *nadV* gene (1339 bp) belonging to APP biovar 2. Moreover, two of the biovar 2 samples were assigned to serotype 2; in the other two samples, the *apxIVA* gene fragment was only detected, and no specific serotype was assigned to it.

## 4. Discussion

This study aimed to investigate the dissemination of APP serotypes in Piedmontese pig farms (Italy). Specifically, the identification of APP infection was performed on lungs collected from deceased pigs reported by farmers and veterinarians as suspected cases of PPP. To our best knowledge of the scientific literature, there has been no exploration of APP prevalence and serotype distribution in Italy, and limited published information exists regarding the circulating APP serotypes. Monitoring lung lesions in pigs is an essential tool for assessing risk factors on farms and implementing prevention or control measures. However, abattoir examinations can be challenging due to the difficulties in record-keeping, execution speed, and the difficulties in obtaining an accurate etiological diagnosis. On the contrary, an often-underestimated control point for the surveillance of the pathologies, and consequently of the dead, is the farm itself. In this study, we subjected the lungs collected to different scoring systems. Among several scoring systems for assessing respiratory lesions, SPES is extensively employed to quantify pleurisy. In this study, SPES evaluation reported most samples with a score 0 (56/107 samples 52.3%). However, samples with a score 0 according to SPES may have lesions other than pleurisy (i.e., catarrhal bronchopneumonia or interstitial pneumonia), as observed in this study by the second score method applied. Our study shows an underestimation of APP infections by the SPES system, considering the high number of samples with a score 0 but, on the contrary, with positive APP identification. This study reported a different situation in pig farms caused by APP infections in comparison with other Italian studies investigating pulmonary lesions at slaughterhouses [20,21]. The literature shows lower SPES scores (from a minimum of 0.79 to a maximum of 0.97) for the investigated carcasses than that of our study, which reports an average SPES value of 1.74. The in-field investigation reported in this study highlights a strongly different situation with a higher prevalence of APP infection and more severe lesions than the slaughterhouse scenario. For a comprehensive pathological understanding, hematoxylin and eosin (HE) histopathology was also conducted. Microscopic lesions observed included mixed inflammatory infiltrates, congestion, hemorrhage, and pleural thickening, consistent with both macroscopic results and histopathological descriptions in the literature [22].

Furthermore, two approaches have been used to characterize the serotypes APP circulating in Italy and to improve our understanding of the severity of the related respiratory lesions: a culture-dependent method and a culture-free APP DNA identification method. The two approaches yielded notable differences in results, with APP isolated by bacteriological culture in 20/107 (18.7%) samples, while the apxIVA gene was identified in 53/107 (49.5%) lung swab samples. The difficulty in culturing APP likely contributes to this marked difference. Although the identification of APP DNA without isolation should not rule out the presence of an end-phase infection, the histological evaluation confirmed the presence of bacteria in some samples. In general, other studies already demonstrated the possibility of detecting APP without culturing [12].

In this study, the subsequent application of two multiplex PCR protocols facilitated the identification of APP serotypes and biovars. The predominant serotypes were identified as serotypes 2 and 6, followed by serotypes 9/11 and 5. Notably, no official or published data on APP serotypes in Italy were available before this study. However, similar epidemiological investigations have been conducted in European countries: Hungary [23,24], Spain [25], Germany [26], Czech Republic [27,28], and UK [29]. Serotype 2 was the most prevalent in this study, aligning with findings from Hungary and Germany [24,26], while serotype 6 was more predominant in the UK and, to a lesser extent, in Germany [26,29]. A comparison with European countries revealed a variation in serotype distribution, with serotype 2 being predominant in Germany, Belgium, Hungary, the Netherlands, and Denmark. In contrast, serotypes 8 and 7 are the main circulating serotypes in the UK and Spain. Beyond Europe, epidemiological surveys in Canada identified serotype 5 as one of the most frequent [30,31], but only three samples were positive for serotype 5 in this study. Although only five of the 19 described APP serotypes were identified in this initial Italian study, a similar reduction in serotype diversity was observed in the later years of the German study [26].

Additionally, this study reported the first-ever putative co-infection by three different APP serotypes, a novel insight facilitated by the application of a culture-free APP DNA-targeting approach. In all the other mentioned studies, only a culture-dependent method was adopted, and serotype identification was conducted only on isolated strains. In addition, in this study, four non-typable APP serotypes have been observed. Similarly, non-typable APP serotypes have been detected in previous studies [26,29,30] and potentially may represent new serotypes different from the ones already known. This finding can be supported by the continuing evolution and diversification of APP over time and the proposal of new serotypes year after year [9,32]. In particular, the main causes of non-typable APP strain findings have been demonstrated to be either the results of insertion phenomena on the cps locus coding for the surface carbohydrates [33] or the identification of serotypes never characterized before [32,34]. Moreover, it is not possible to exclude the possibility of a lack of amplification during PCR due to sequence modification at primer sites [9,35]. Furthermore, four samples were positive for APP biovar 2 in Piedmont, similar to Germany, but different from Spain, where biovar 2 is predominant [25,26]. Generally considered less virulent than biovar 1 due to the absence of specific apx toxins production [8], the presence of biovar 2 in Piedmont aligns with the findings in Germany. However, very limited information is available on the prevalence of APP biovars.

Overall, according to Italian customs, routine veterinary diagnostic laboratories still primarily rely on culture methods to isolate APP, followed by species identification using MALDI-TOF MS. Moreover, APP serotype identification is rarely performed for diagnostic purposes, despite its potential utility in better defining vaccination strategies at the regional or national level. Notably, the implementation of a culture-free multiplex PCR approach for APP serotype identification has demonstrated enhanced efficacy compared to conventional culture methods and could be regularly adopted for diagnostic purposes.

## 5. Conclusions

In this study, the circulation of APP serotypes was explored for the first time in Italy and correlated with both macroscopic and microscopic lesions. The most frequently identified serotypes were serotypes 2 and 6.

The novelty of this study lies in leveraging pig farms as an observation and sampling center for lung diseases, thereby initiating efforts to address gaps in understanding the circulation of APP serotypes in Italy. This study also indicates the added value of an integrated diagnostic approach, including macroscopic and microscopic analysis, microbiological analysis, and PCR, as a tool applied in field conditions.

## Figures and Tables

**Table 1:** Gross scoring system results for lobe involvement evaluation. Results are expressed as mean value ± standard deviation (sd).

Pulmonary Lobe	Score (Mean ± sd)
Left cranial	1.99 ± 1.41
Left medium	2.38 ± 1.4
Left caudal	2.17 ± 1.2
Right cranial	2.2 ± 1.32
Right medium	2.64 ± 1.32
Right accessory	2.42 ± 1.52
Right caudal	2.26 ± 1.22

**Table 2:** Microbiological results were obtained in 107 samples of lungs collected in the present study.

Microorganisms	Number of Infected Individuals (%)
Porcine reproductive and respiratory syndrome virus (PRRSV)	31 (29%)
*Mesomycoplasma hyopneumoniae*	11 (10.3%)
*Escherichia coli*	10 (9.3%)
*Streptococcus suis*	10 (9.3%)
*Pasteurella multocida*	9 (8.4%)
*Actinobacillus lignieresii*	5 (4.7%)
*Trueperella pyogenes*	4 (3.7%)
*Bordetella bronchiseptica*	3 (2.8%)
*Staphylococcus aureus*	3 (2.8%)
*Staphylococcus* spp.	1 (1.7%)
*Escherichia* spp.	1 (1.7%)
*Swine Influenza Virus*	1 (1.7%)

## Data Availability

All data presented in this study are available directly in the article or in the Supplementary Files. All other relevant information can be obtained from the corresponding author upon request.

## Supplementary Materials

The following additional files are available online at: https://www.mdpi.com/article/10.3390/ani14152255/s1, Figure S1: PCR testing; Table S1: Additional histopathological lesions of 107 examined lungs.
