# Supplementary material for: Actinobacillus pleuropneumoniae Serotypes by Multiplex PCR Identification and Evaluation of Lung Lesions in Pigs from Piedmont (Italy) Farms
Source: Animals (Basel). 2024 Aug 3;14(15):2255. doi: 10.3390/ani14152255 (PMC11311043; doi:10.3390/ani14152255)
Supplement: Supplementary file 1 [file animals-14-02255-s001.zip › animals-3046394-supplementary.pdf]

Table S1. Additional histopathological lesions of 107 examined lungs.

Microscopic lesions	No. of positive samples (%)
Alveolar oedema	41 (38.3%)
Hyperplastic lymphoid follicles	30 (28%)
Lymphocytic infiltrate	22 (20.6%)
Proliferation of the bronchiolar epithelium	19 (17.7%)
Interalveolar septa thickening	16 (14.9%)
Granulocytic infiltrate	9 (8.4%)
Bacterial colonies	9 (8.4%)
Emphysema	7 (6.5%)
Necrosis	5 (4.7%)
Atelectasis	4 (3.7%)
Parenchymal consolidation	4 (3.7%)
Eosinophilic infiltrate	2 (1.9%)
Vascular thrombosis	1 (0.9%)
Fungal hyphae	1 (0.9%)
Parasitic lesions	1 (0.9%)
