# Supplementary figures and images for: Actinobacillus pleuropneumoniae Serotypes by Multiplex PCR Identification and Evaluation of Lung Lesions in Pigs from Piedmont (Italy) Farms
Source: Animals (Basel). 2024 Aug 3;14(15):2255. doi: 10.3390/ani14152255 (PMC11311043; doi:10.3390/ani14152255)

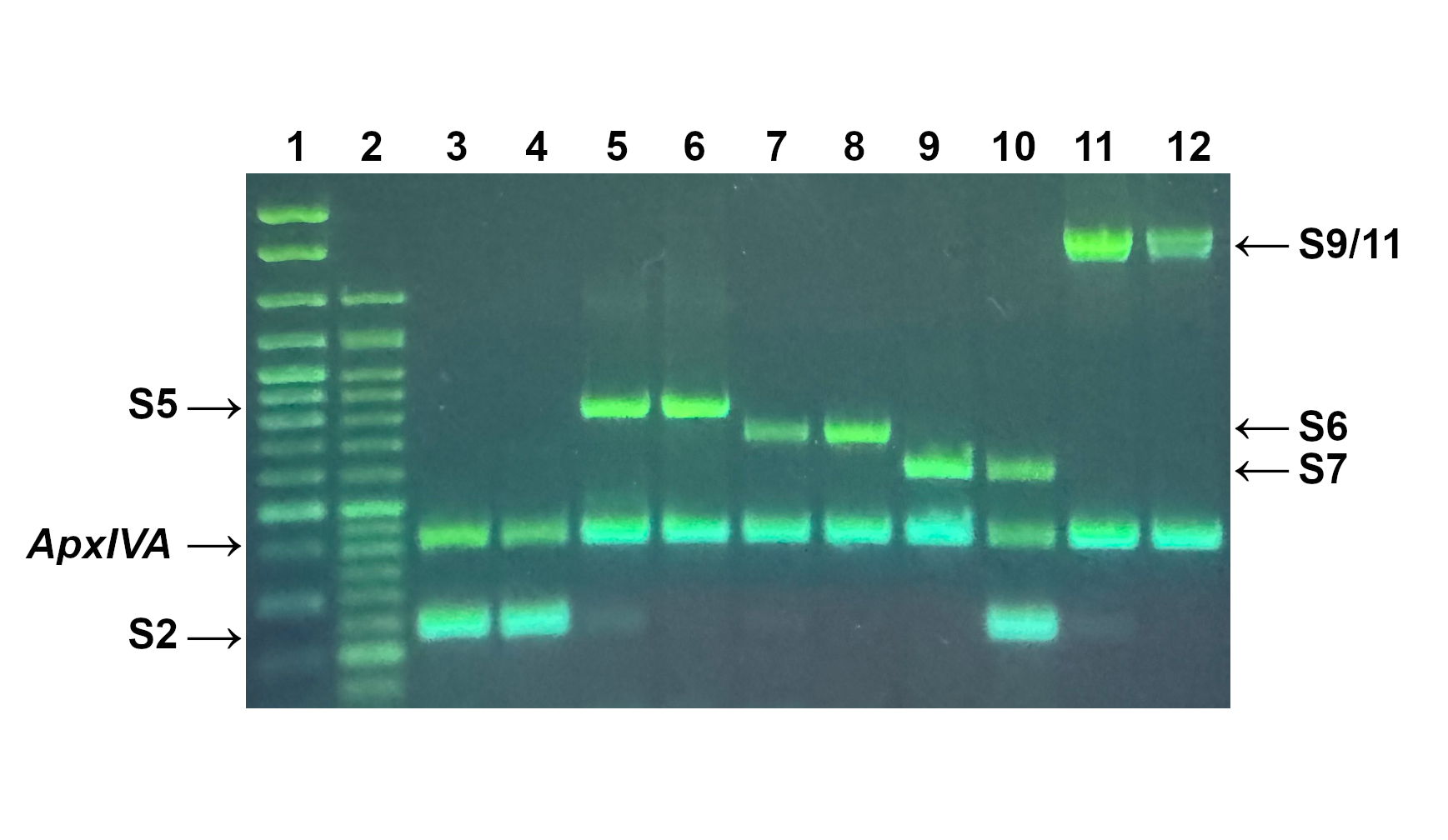

Supplement: Supplementary file 1 [file animals-14-02255-s001.zip › animals-3046394-supplementary.tif]
